# Prediction of the Auto-Ignition Temperatures of Binary Miscible Liquid Mixtures from Molecular Structures

**DOI:** 10.3390/ijms20092084

**Published:** 2019-04-27

**Authors:** Shijing Shen, Yong Pan, Xianke Ji, Yuqing Ni, Juncheng Jiang

**Affiliations:** Jiangsu Key Laboratory of Hazardous Chemicals Safety and Control, College of Safety Science and Engineering, Nanjing Tech University, Nanjing 210009, China; ShijingShen_njtech@163.com (S.S.); 1090997173@njtech.edu.cn (X.J.); nyqforwork@outlook.com (Y.N.); ypnjut@126.com (J.J.)

**Keywords:** quantitative structure-property relationship (QSPR), auto-ignition temperature (AIT), simplex representation of molecular structure (SiRMS), binary miscible liquid mixtures

## Abstract

A quantitative structure-property relationship (QSPR) study is performed to predict the auto-ignition temperatures (AITs) of binary liquid mixtures based on their molecular structures. The Simplex Representation of Molecular Structure (SiRMS) methodology was employed to describe the structure characteristics of a series of 132 binary miscible liquid mixtures. The most rigorous “compounds out” strategy was employed to divide the dataset into the training set and test set. The genetic algorithm (GA) combined with multiple linear regression (MLR) was used to select the best subset of SiRMS descriptors, which significantly contributes to the AITs of binary liquid mixtures. The result is a multilinear model with six parameters. Various strategies were employed to validate the developed model, and the results showed that the model has satisfactory robustness and predictivity. Furthermore, the applicability domain (AD) of the model was defined. The developed model could be considered as a new way to reliably predict the AITs of existing or new binary miscible liquid mixtures, belonging to its AD.

## 1. Introduction

The auto-ignition temperature (AIT) is defined as the lowest temperature at which the substance spontaneously ignites in ambient air, without an external ignition source, such as a spark or flame. AIT is one of the most important parameters applied to classify the chemicals based on their degree of flammability [1]. The experimental AIT values are the main source of the AIT data used in production. However, the measurement of AITs is expensive and time-consuming. Especially for the mixtures, the measurement is more difficult, since the AITs of the mixtures are closely related to their compositions and ratios, which are rather difficult to test one-by-one. Therefore, it is of great significance to develop theoretical models for predicting the AITs of mixtures.

Many theoretical models for predicting the AITs of pure flammable liquids have been proposed [2,3,4,5]. However, only a few efforts have been made to predict the AITs of mixtures. Rota et al. [6] developed a kinetic model to predict the AITs of 46 gas mixtures, including ammonia, hydrogen, methane, and air at high pressure and temperature. The average relative error (ARE) and the average absolute error (AAE) of the proposed model were about 3.5% and 25 K, respectively. Peper et al. [7] proposed a simple weighting function formula to predict the AITs of polyurethane raw material mixtures. However, the results showed that the calculated values of AITs of five mixtures are 20 K higher than the measured values. Lan et al. [8] presented a zero-dimensional model to predict the AITs of 24 binary miscible liquid mixtures. Due to the lack of a chemical kinetic mechanism, the predictive ability of the model for higher hydrocarbons is poor.

The quantitative structure-property relationship (QSPR) method, as a mathematical method, relates the properties of interest to the molecular structures of chemicals. It can be expected to capture the relationships between the molecular structures and desired properties without detailed knowledge of the mechanisms of interaction. In addition, QSPR is considered to be a time-saving and effective method for prediction of the desired properties. In recent years, several QSPR models have been successfully developed to predict the physicochemical properties of mixtures, such as toxicity, boiling point, flash point, and critical parameters, all of which showed satisfactory stability and predictivity [9,10,11,12,13].

The most challenging problem in QSPR studies for mixtures is the representation of structure characteristics of mixtures. There are several different descriptor types for mixtures reported in the literature: descriptors based on the partition coefficient for a mixture, integral additive descriptors, integral non-additive descriptors of mixtures, and fragment non-additive descriptors [14]. As one of the typical fragment non-additive descriptors, Simplex Representation of Molecular Structure (SiRMS) descriptors can be theoretically applied to any investigated activity or property, and could capture the interaction or joint effect of components. Recently, SiRMS descriptors have been successfully employed in QSPR studies for mixtures [15,16,17].

In this work, for the first time, the QSPR method is applied to study the quantitative relationships between the molecular structures and AITs of binary miscible liquid mixtures. The main purpose of this study is to develop a new method for predicting the AITs of binary miscible liquid mixtures, including: (i) development of SiRMS descriptors for mixtures; (ii) establishment of a QSPR model for the AITs of binary miscible liquid mixtures; (iii) rigorous internal and external model validations; and (iv) definition of the model applicability domain (AD).

## 2. Results and Discussion

### 2.1. Results of Prediction

According to the “Compounds out” strategy, the dataset is divided into a training set with 99 mixtures and a test set with 33 mixtures. By performing the GA-MLR procedure on the training set, starting with the calculated 434 simplex descriptors, a best subset of six descriptors was obtained. The definitions and types of these selected descriptors are shown in Table 1. The corresponding best model is presented as follows:

(1) $$\mathrm{AIT}\;=\;700.630\;+\;36.735X_{1}\;-\;56.130X_{2}\;+\;70.943X_{3}\;+\;52.446X_{4}\;-\;111.781X_{5}\;-\;92.718X_{6}\;\mathrm{range}:\;496.15\;K\;\leq \;\mathrm{AIT}\;\leq \;798.15\;K\;R^{2}\;=\;0.958,\;{Q^{2}}_{\mathrm{LOO}}\;=\;0.950,\;s=\;15.411,\;F=\;345.869,\;n=99$$

where *n* is the number of mixtures in the training set, *s* is the standard error of the model, and *F* is the Fischer F-ratio.

Moreover, the relative significance and contribution of each descriptor on the AIT were determined by the mean effect (ME) analysis, which is calculated as follows:

(2) $${\mathrm{ME}}_{j}=\frac{\beta_{j}\sum_{i=1}^{i=n}d_{ij}}{\sum_{j}^{m}\beta_{j}\sum_{j}^{n}d_{ij}}$$

where ${\mathrm{ME}}_{j}$ represents the mean effect for the descriptor *j*, $\beta_{j}$ is the coefficient of the descriptor *j*, $d_{ij}$ is the value of the descriptors of interest for each mixture, *m* is the number of descriptors in the model, and *n* is the number of dataset members. The symbol (positive or negative) of ME represents the trend of the impact of each descriptor on the AIT. The greater the absolute value of the coefficient is, the more important the descriptor is.

As can be concluded from Table 2, the |S|n|||4|||REFRACTIVITY|B-B-B-B descriptor has the greatest influence on AIT. In addition, the relative importance and contribution of each descriptor in the model was determined and ranked as follows based on the ME values: |S|n|||4|||REFRACTIVITY|B-B-B-B > |S|n|||4|||CHARGE|A.A-A-B > |M|n|||4|||REFRACTIVITY|B-B.B-C > |S|n|||4|||elm|C-C(-C)=O > |M|n|||4|||CHARGE|A-A.B-C > |S|n|||4|||elm|C-C(-O)=O.

**Figure 1:**
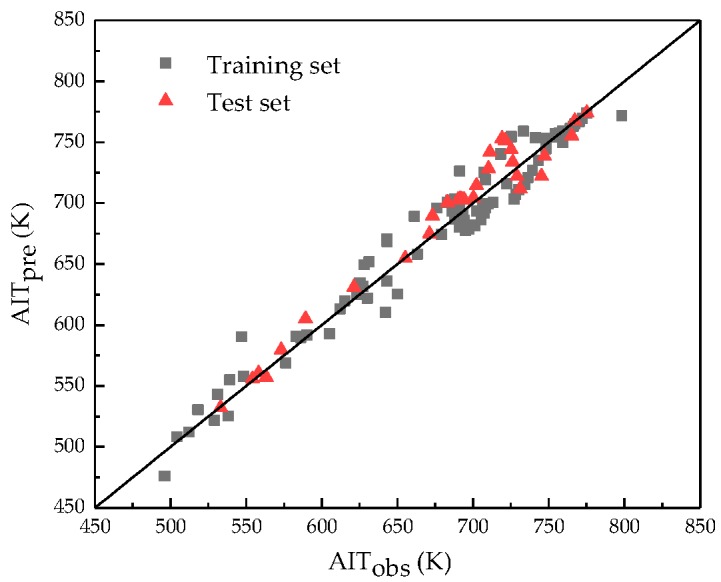
Correlation between the predicted and observed AIT values for both the training and test sets.

**Figure 2:**
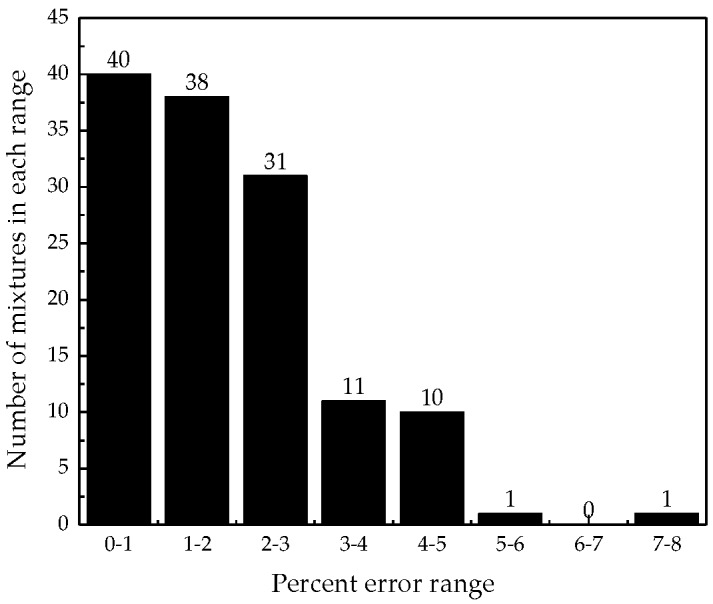
The percent errors obtained by the presented model and the number of mixtures in each range.

The developed model was then employed to predict the AIT values of mixtures in the test set for external validation. The predicted AIT values are presented in the Supplementary Table S1. The main statistical parameters of the model are presented in Table 2. As can be seen from Table 3, the AAE and RMSE values were as low as possible, which indicated that the presented model has acceptable predictive capability. A plot of the predicted AIT values versus the observed ones for both the training and test sets is presented in Figure 1. Thus, this showed a reasonable agreement between the predicted and observed AIT values across the whole dataset. The predicted percentage error of all the 132 mixtures was also calculated, which is shown in Figure 2. The obtained average percentage error for these mixtures was 1.8% and the maximum percentage error was 7.9%.

### 2.2. Model Stability Validation and Results Analysis

**Figure 3:**
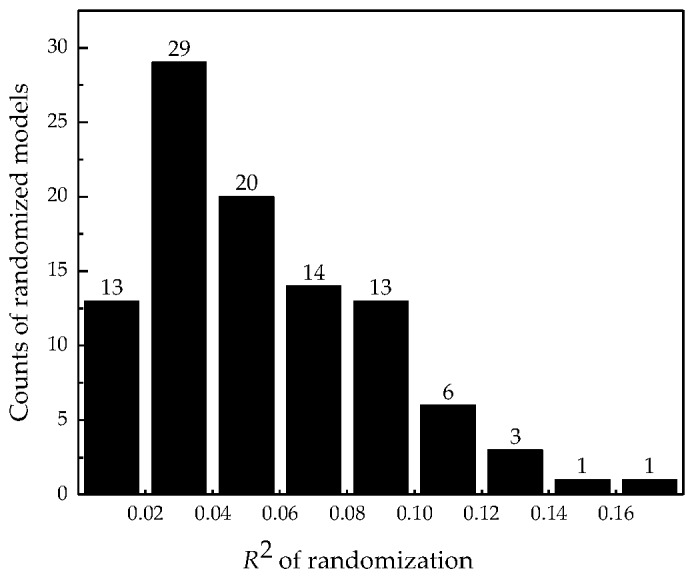
Histogram of *R*^2^ of randomization versus frequency of occurrence of the randomized models.

In this study, the Y-randomization test was performed on the training set 100 times. The obtained *R*^2^ of randomization versus the frequency of occurrence of the randomized models are presented in Figure 3. The resulting maximum, minimum, and average values of the achieved highest random *R*^2^ were 0.173, 0.004, and 0.055, respectively, while the value of SD was 0.035. The difference between the *R*^2^ of the original MLR model and the mhr *R*^2^ is higher than 3 SD. It can be concluded that there is no chance correlation in the proposed model.

**Figure 4:**
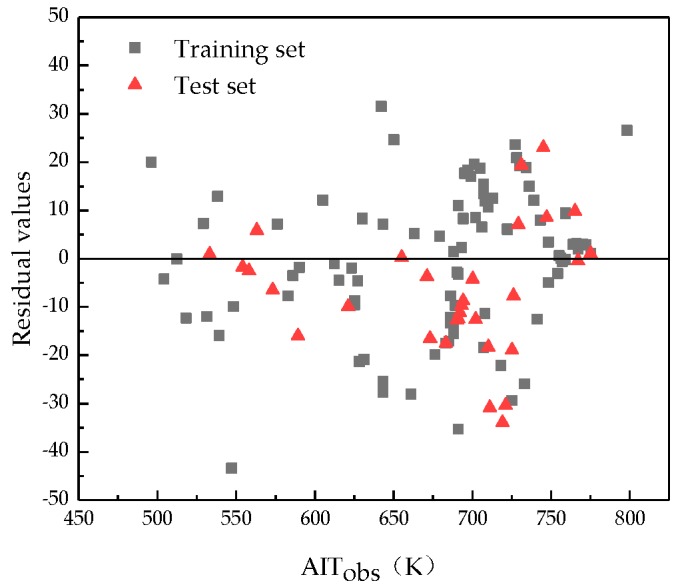
Plot of the residuals versus the observed AIT values for the MLR model.

The predicted residuals and observed values for the developed model are shown in Figure 4. It can be seen that the calculated residuals are randomly distributed on both sides of the zero baseline, which demonstrates that no systematic errors exist in the proposed model.

From all of the above validation results, it can reasonably be concluded that the proposed MLR model has satisfactory robustness and predictability. So, it can be reliably and conveniently employed to predict the AITs of binary miscible liquid mixtures, solely from their molecular structures and mole fractions.

### 2.3. Applicability Domain of the Proposed Model

**Figure 5:**
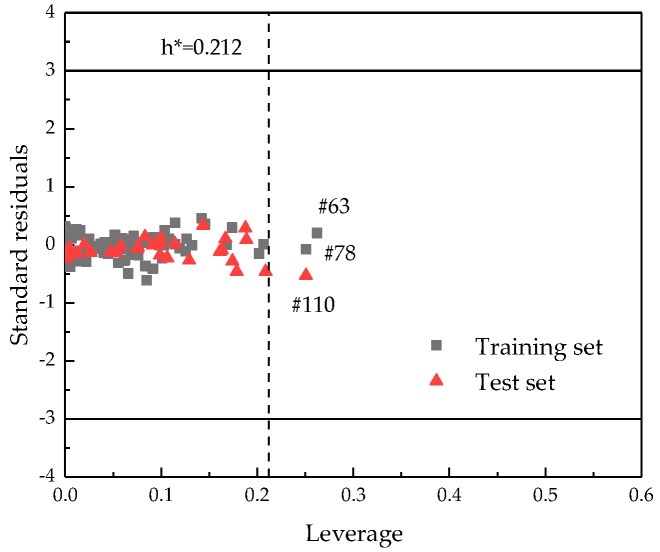
A Williams plot describing the applicability domain of the Quantitative Structure-Property Relationship (QSPR) model (h* = 0.212).

A Williams plot for the proposed QSPR model is shown in Figure 5. The AD is established inside a squared area within ±3 standard deviations and a leverage threshold h* of 0.212. In Figure 5, there are three possible outliers (namely, #63, #78, and #110) in the dataset with higher leverage values (*h* > h*). The structures of these mixtures are obviously different from the others. However, their AITs can still be satisfactorily predicted by the present model within the standard deviation. Thus, the predictions are considered to be acceptable. Therefore, the developed model can be expected to reliably predict the AITs for the binary miscible liquid mixtures falling within the corresponding applicability ranges. However, it should be stated that there is also a limitation to the AD of the model in terms of chemical diversity, since the studied dataset only contained 10 different pure compounds. However, it is rather difficult to find a further larger set of AIT data for binary mixtures in the open literature that contains more and different pure compounds.

## 3. Materials and Methods

### 3.1. Dataset

The dataset consists of 132 binary miscible liquid mixtures and originates from Lan et al.’s work [8], the detail of which can be found in the Supplementary Table S1. The pure compound components include alcohols, acids, esters, benzenes, ketones, and alkanes. All of the AIT values were obtained by experimental tests according to the ASTM E659-78 test standard (American Society for Testing and Materials). The AIT values of the whole dataset range from 496.15 K to 798.15 K. As is well-known, with a larger dataset, a better predictive model could be developed; however, it is rather difficult to find a larger set of AIT data for binary mixtures in the open literature in terms of chemical diversity.

### 3.2. Descriptor Calculation and Reduction

An important step in a QSPR study is the characterization of the molecular structures. In this study, the binary mixtures were represented by a variety of SiRMS descriptors. In the framework of SiRMS, any molecule can be represented as a system of different fragments (simplexes) of fixed composition, structure, chirality, and symmetry simplexes [18]. All of the possible topological structure types of simplexes are shown in Table 3.

Bounded and unbounded two-dimensional (2D) simplexes were used. Bounded simplexes were used to describe pure compounds, while unbounded simplexes can describe both the pure compounds and the mixtures. Thus, during descriptor generation, a special mark is used to distinguish them. The details of the procedure for calculation of 2D simplex descriptors for mixtures in this study are as follows. Firstly, the 2D chemical structures of each pure substance were drawn in MarvinSketch (version 15.6.29.0, ChemAxon, Budapest, Hungary) [19], and optimized based on the “clean in 2D” method by this software. For binary mixtures, the program generated the simplexes of individual species and mixture simplexes with atoms from two compounds. Then, each atom of the fragment obtained a calculated value by the *cxcalc* tool [19] and the atoms were divided into the corresponding groups: (i) partial charge A ≤ −0.05 < B ≤ 0 < C ≤ 0.05 < D; (ii) lipophilicity A ≤ −0.5 < B ≤ 0 < C ≤ 0.5 < D; and (iii) refraction A ≤ 1.5 < B ≤ 3 < C ≤ 8 < D. Three characteristics of atom H-bond formation ability were specified: A (acceptor of hydrogen in H-bond), D (donor of hydrogen in H-bond), and I (indifferent atom). In this work, fragments with four atoms were considered to reduce the probability of the model over-fitting and ensure its predictivity and AD [20]. The described SiRMS descriptors can be implemented in the open-source software (version 1.1.2, GitHub, San Francisco, California, America) [21] written on Python 3, which is available on the Github repository.

Descriptors of constituent parts (compounds 1 and 2) are weighted according to their molar fraction, which was calculated as follows:

(3) $$D_{s}{\;=\;x}_{1}D_{1}{\;+\;x}_{2}D_{2}.$$

Meanwhile, mixture descriptors are multiplied on the doubled minimal weight according to Equation (4).

(4) $$D_{M\;}{=\;2x}_{1}D_{1+2}$$

where *x*_1_ and *x*_2_ are molar fractions of compounds 1 and 2 (*x*_1_ < *x*_2_ and *x*_1_ + *x*_2_ = 1), respectively, and *D*_1_, *D*_2_, and *D*_1+2_ are descriptor values for individual compounds 1 and 2, and for their mixtures, respectively. Furthermore, the volume ratio obtained from the literature [8] needs to be converted to a molar ratio first, since the calculation rules are based on the molar ratio.

A concatenation of D_S_ and D_M_ represents the mixture descriptors of the whole dataset. Finally, a total set of 434 simplex descriptors was achieved.

### 3.3. Descriptor Selection and Model Development

The key step in QSPR modeling is to find the optimal descriptors that make a significant contribution to the AITs of binary miscible liquid mixtures. The well-known genetic algorithm (GA) is a powerful optimization method to solve this problem and has been successfully applied to feature selection in previous QSPR studies [22,23,24]. In this study, genetic algorithm along with multiple linear regression (GA-MLR) was used to find the optimal subset that accurately represented the relationships between molecular structures and AITs of binary liquid mixtures. The GA-MLR was performed by the MATLAB M-file written in our laboratory. The fitness function of this method corresponds to the root mean square error of cross-validation (rmsecv).

The selection program is started with one descriptor, and the best one-parameter regression model, with the minimal rmsecv value, should be obtained. Then, the number of desired variables should be increased to two, three, four, etc. and the corresponding best multi-parameter regression models with the desired number of descriptors should be found. When the number of descriptors was increased and the rmsecv did not significantly improve, it can be determined that the optimum subset of descriptors that produce the best MLR model has been achieved [25].

### 3.4. Model Validation

Model validation is a necessary step to ensure the reliability of the developed QSPR models. In this study, both internal and external validation methods were employed to validate the developed QSPR model.

Cross-validation (CV) is one of the most common methods for internal validation. A good CV result often represents a good robustness and high internal predictive capability of QSPR models. In this study, leave-one-out (LOO) cross-validation (*Q*^2^_LOO_) was employed, which is calculated with the following equation:

(5) $$Q_{\mathrm{LOO}}^{2}=1-\frac{\sum_{i=1}^{\mathrm{training}}{\left(y_{i}-y_{0}\right)}^{2}}{\sum_{i=1}^{\mathrm{training}}{\left(y_{i}-\bar{y}\right)}^{2}}$$

where *y*_i_, *y*_0_, and $\bar{y}$ are respectively the observed, predicted, and mean observed AIT values of the mixtures in the training set.

External validation is significant and necessary to determine both the predictive capability and generalizability of a developed model for new mixtures. There are three widely used strategies, including “Points out”, “Mixtures out”, and “Compounds out” for dataset partition. Among these three strategies, the “Compounds out” strategy is the most rigorous one and it will fully reflect the ability of models to predict mixtures with a new compound [14,17]. Thus, in this study, the external validation was carried out by randomly splitting the available dataset into a training set (75% of the dataset), and an external test set (25% of the dataset) based on the “Compounds out” partition strategy. The training set is used for descriptor selection and model development, while the test set is used for model validation. The predictive capability of a QSPR model can be judged by an external *Q*^2^_EXT_, which is defined as follows:

(6) $$Q_{\mathrm{EXT}}^{2}=1-\frac{\sum_{i=1}^{\mathrm{test}}{\left(y_{i}-y_{0}\right)}^{2}}{\sum_{i=1}^{\mathrm{test}}{\left(y_{i}-{\bar{y}}_{\mathrm{tr}}\right)}^{2}}$$

where *y*_i_ and *y*_0_ are the observed and predicted AIT values of the mixtures in the test set, respectively, and ${\bar{y}}_{\mathrm{tr}}$ is the mean observed AIT values of the mixtures in the training set.

Additionally, a Y-randomization test was employed to further ensure the robustness of the model. The dependent-variable vector (Y vector) was scrambled randomly, while all independent data variables were unchanged, and the robustness of the developed model was tested. The process was repeated 50–100 times. In each model, the highest *R*^2^ value obtained by descriptor selection is recorded as the highest random *R*^2^ of randomization. In addition, the mean highest random (mhr) *R*^2^ and its standard deviation (SD) were calculated by averaging over the repetitions. If all *R*^2^ values of the randomized models are lower than that of the original model, and the difference between *R*^2^ of the original model and mhr *R*^2^ is higher than 2.3 SD for significance at the 1% level, then higher than 3 SD for the 0.1% level. It can be concluded that there is no chance correlation in the model development, and the model can be considered as an acceptable model [26].

The squared correlation coefficient (*R*^2^) is used to determine the calibration capability of the model. The average absolute error (AAE) and root mean square error (RMSE) were employed to evaluate the predictive capability of the developed models, which are calculated as follows:

(7) $$\mathrm{AAE}\;=\;\frac{\sum_{i\;=\;1}^{n}\;\left|y_{i}{-y}_{0}\right|}{n}$$

(8) $$\mathrm{RMSE}\;=\sqrt{\frac{\sum_{\;i=\;1}^{n}{\left(y_{i}{-y}_{0}\right)}^{2}}{n}}$$

where *y*_i_ is the observed value, *y*_0_ is the predicted value, and *n* is the number of mixtures in the dataset.

### 3.5. Applicability Domain

According to Organization for Economic Cooperation and Development (OECD) principle 3 [27], the AD should be defined once a QSPR model is obtained. The AD of the model is a theoretical region of the chemical space, which is defined by the descriptors and modeled response. Statistical models can provide reliable predictions for the mixtures in this region. If all of the AIT values are within the AD range, it can be considered that the model is reliable. In this study, the Williams plot was depicted to analyze the AD.

For the *x*-axis, the leverage value (*h_i_*) describes the impacts of the objects on the model, which is defined as:

(9) $$h_{i}{\;=\;x}_{i}{\left(X^{T}X\right)}^{-1}x_{i}^{T},$$

where *x_i_* is the descriptor column-vector of the considered mixtures and *X* is the descriptor matrix derived from the training set descriptor values. The warning leverage value (h*) is calculated as follows:

(10) $$h*=\frac{3\left(p+1\right)}{n}$$

where *p* is the number of model parameters and *n* is the number of training mixtures.

If the *h*_i_ of a mixture is greater than the h*, it can be considered as outside of the AD range of the model. For the *y*-axis, a Williams plot presented the Euclidean distances of the mixtures to the model measured by the cross-validated standardized residuals. The mixture is classified as an outlier when the cross-validated standardized residual is greater than 3 standard deviation units.

## 4. Conclusions

In this work, for the first time, a QSPR model has been developed for predicting the AITs of binary miscible liquid mixtures from the molecular structures. To the best of our knowledge, the largest existing database of AITs for binary mixtures was employed for modeling. The most rigorous “compounds out” method was used to divide the training set and the test set. The SiRMS methodology was employed to describe the structure characteristics of binary mixtures. The best-resulted QSPR model was a six-parameter linear equation. The model validation results showed the satisfactory robustness and predictivity of the model. The developed model would be expected to provide a new way to reliably predict the AIT values of existing or new binary miscible liquid mixtures, belonging to their AD. Furthermore, the method provides some guidance for prioritizing the design of safer liquid mixtures with desired properties.

## Figures and Tables

**Table 1:** Descriptors selected in the presented model for prediction of the Auto-ignition Temperature (AIT).

Symbol	Descriptor	Definition	Type	Mixing Rule	ME Value
X_1_	|S|n|||4|||CHARGE|A.A-A-B	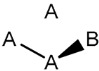	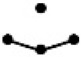	$x_{1}D_{1}{\;+\;x}_{2}D_{2}$	−66.821
X_2_	|S|n|||4|||REFRACTIVITY|B-B-B-B	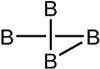	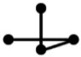	$x_{1}D_{1\;}{+\;x}_{2}D_{2}$	155.161
X_3_	|S|n|||4|||elm|C-C(-C)=O	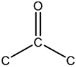	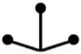	$x_{1}D_{1}{+x}_{2}D_{2}$	−54.633
X_4_	|S|n|||4|||elm|C-C(-O)=O	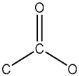	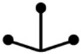	$x_{1}D_{1}{+x}_{2}D_{2}$	−14.773
X_5_	|M|n|||4|||CHARGE|A-A.B-C	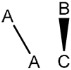	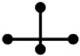	${2x}_{1}D_{1+2}$	21.835
X_6_	|M|n|||4|||REFRACTIVITY|B-B.B-C	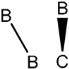	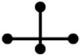	${2x}_{1}D_{1+2}$	59.231

**Table 2:** The main statistical parameters of the obtained Multiple Linear Regression (MLR) model.

Statistical Parameters	Training Set	Test Set
*R* ^2^	0.958	0.942
*Q* ^2^_LOO_	0.950	-
*Q* ^2^_EXT_	-	0.942
*RMSE*	15.333	15.740
*AAE*	12.395	12.531
*ARE*	1.9%	1.8%
*n*	99	33

**Table 3:** Basic types of simplexes.

Basic Type	1	2	3	4	5	6	7	8	9	10	11
simplex	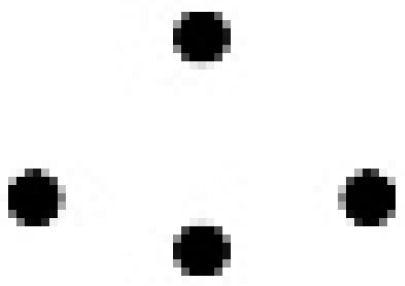	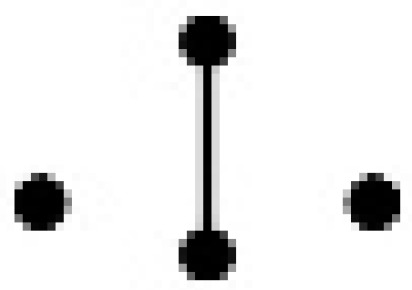	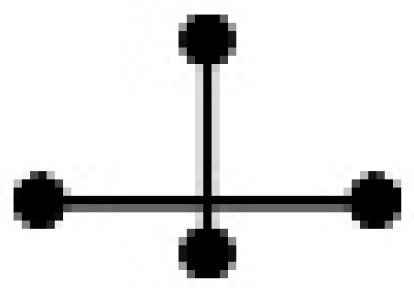	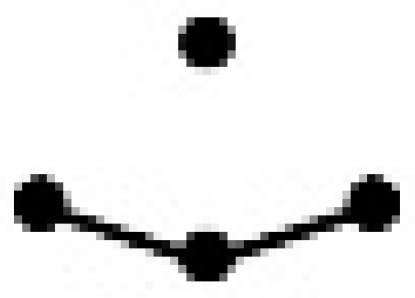	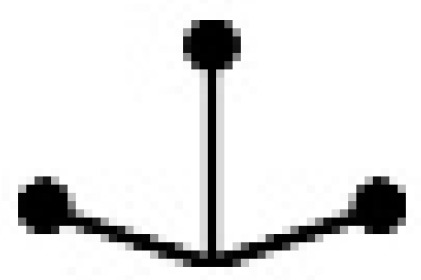	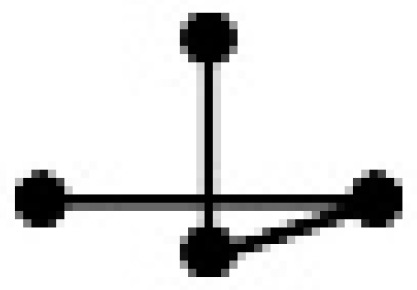	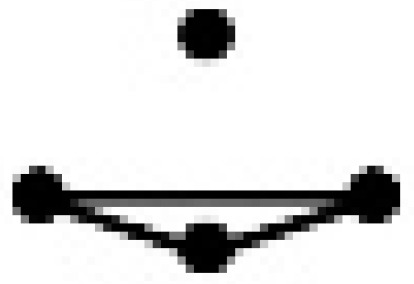	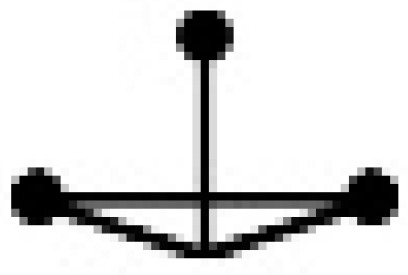	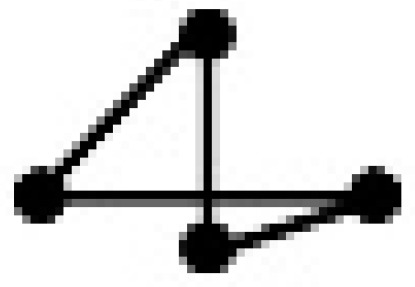	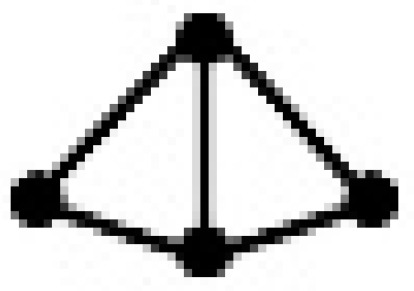	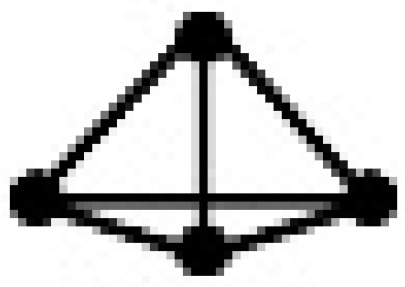

## Supplementary Materials

Supplementary materials can be found at https://www.mdpi.com/1422-0067/20/9/2084/s1. Table S1: A complete list of the compositions of the 132 binary miscible liquid mixtures and their predicted and observed AIT values, as well as the values of the six employed SiRMS descriptors in the model.

## Abbreviations

QSPR	Quantitative structure-property relationship
AIT	Auto-ignition temperature
SiRMS	Simplex representation of molecular structure
GA	Genetic algorithm
MLR	Multiple linear regression
AD	Applicability domain
ARE	Average relative error
AAE	Average absolute error
GA-MLR	Genetic algorithm along with multiple linear regression
rmsecv	root mean square error of cross-validation
CV	Cross-validation
Q^2^_LOO_	Leave-one-out cross-validation
mhr	mean highest random
RMSE	Root mean square error
hi	Leverage value
*h**	Warning leverage value
ME	Mean effect
SD	Standard deviation
